# Prevalence of Delayed Insulin Initiation by Age at Diagnosis in US Adults with Type 1 Diabetes

**DOI:** 10.1007/s11606-025-09546-y

**Published:** 2025-04-25

**Authors:** Anna R. Kahkoska, Sui Zhang, Justin B. Echouffo-Tcheugui, Elizabeth Selvin, Michael Fang

**Affiliations:** 1 Department of Nutrition, Gillings School of Global Public Health, University of North Carolina at Chapel Hill, Chapel Hill, NC USA; 2 Division of Endocrinology and Metabolism, School of Medicine, University of North Carolina at Chapel Hill, Chapel Hill, NC USA; 3 Center for Aging and Health, University of North Carolina at Chapel Hill, Chapel Hill, NC USA; 4 Department of Epidemiology, Johns Hopkins Bloomberg School of Public Health, Baltimore, MD USA; 5 Department of Medicine, Division of Endocrinology, Diabetes and Metabolism, Johns Hopkins School of Medicine, Baltimore, MD USA

Although type 1 diabetes has often been considered a disease of childhood, 37% of cases occur after age 30.^1^ Misdiagnosis of adult-onset type 1 diabetes as type 2 diabetes is common and may delay the initiation of insulin.^2^ However, existing studies examining the timing of insulin therapy are based on select clinical populations, limiting generalizability.^3^ Identifying persons with type 1 diabetes in the general population at high risk for delayed insulin initiation is an important public health concern, as delays can increase the risk for short- and long-term complications. Our objective was to characterize the prevalence of delayed insulin initiation in US adults with type 1 diabetes according to age at diagnosis.

The National Health Interview Survey (NHIS) is a nationally representative survey of the noninstitutionalized US population.^4^ We combined all NHIS survey cycles for which diabetes subtype data were available (2016, 2017, 2019, and 2020–2023). The National Center for Health Statistics Institutional Review Board approved the survey protocol. All participants provided written informed consent. NHIS data are publicly available (https://www.cdc.gov/nchs/nhis/documentation/2023-nhis.html). 

Sociodemographic and medical data in the NHIS were self-reported and collected through interviews. The NHIS typically collects data through in-person interviews but relied primarily on telephone interviews from January to April 2021 due to the COVID-19 pandemic*.*

Persons with diabetes reported diagnosed diabetes type (types 1 or 2), current use of insulin, and time from diagnosis to insulin initiation (0–1 months, 1–6 months, 6–12 months, 12 + months).

Consistent with prior studies, we defined type 1 diabetes as a reported diagnosis and current use of insulin.^1^ We defined delayed insulin initiation as starting insulin at least 6 months after diabetes diagnosis.^3^

We included all persons age ≥ 18 years with diagnosed type 1 diabetes. Among these participants, we estimated the percentage who had delayed insulin initiation according to age of diagnosis. Analyses were conducted using Stata 17.0 and used recommended survey weights to generate nationally representative estimates.

The study included 1112 US adults with diagnosed type 1 diabetes (mean age 49; 52% male; 73% non-Hispanic White). The percentage of delayed insulin initiation increased across groups of age at diagnosis with an inflection point with diagnosis at age ~ 30 years (Fig. 1). Up to 26% (approximately 15-25%) of adults diagnosed before 30 had delayed insulin initiation, compared to 48–58% of those diagnosed after age 30. Our results corroborate findings from previous studies showing that older age is associated with a delay in insulin initiation and hospitalization.^3^ Our findings extend existing research by highlighting large differences by age in insulin initiation within a nationally representative population of US adults with type 1 diabetes.

**Figure 1:**
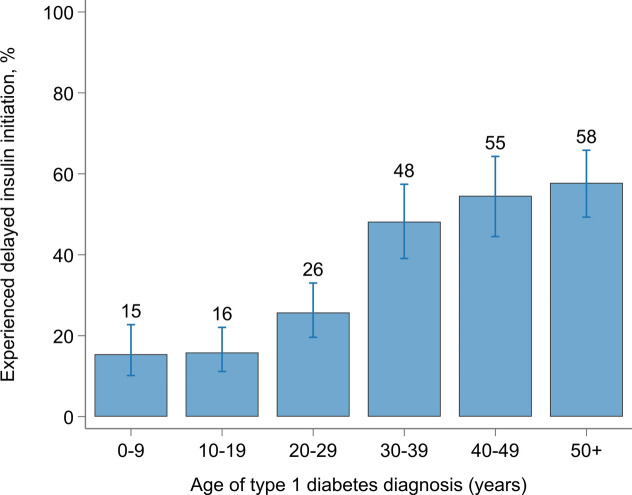
Percentage of US adults with type 1 diabetes experiencing delayed insulin initiation (defined as insulin initiation that occurred more than 6 months after diagnosis), according to self-reported age at type 1 diabetes diagnosis, NHIS 2016–2023. Vertical lines are 95% confidence intervals.

The high prevalence of delayed insulin initiation in adult-onset type 1 diabetes may partially reflect misdiagnosis. Between 30% and 40% of type 1 diabetes cases identified after age 30 are misclassified as type 2 diabetes.^2^ However, the factors underlying misdiagnosis may vary. On one hand, adult-onset type 1 diabetes often is characterized by preserved beta-cell function and therefore presents with mild symptoms that resemble type 2 diabetes. Clinical suspicion of type 1 diabetes may also be lower in adult-onset cases; raising awareness of adult-onset type 1 diabetes is critical for reducing misdiagnosis and ensuring timely and appropriate treatment.^2^ Clinical decision-making tools are being actively developed to aid diagnosis. For example, Leslie et al. have proposed a simple set of questions, termed the “AABBCCs of diabetes classification,” to improve classification of adult-onset diabetes.^2^

Another explanation for the delayed insulin initiation may be a longer “honeymoon phase” in late onset type 1 diabetes, in which case insulin may not be required initially. Unfortunately, there was no way to distinguish whether delayed insulin initiation was related to residual insulin production or misdiagnosis in this study.

The NHIS is limited by the use of self-report information; autoantibody status would be needed to confirm etiologic, autoimmune type 1 diabetes. The results do not represent individuals with undiagnosed type 1 diabetes. Nonetheless, we used a definition of type 1 diabetes that has high accuracy (~ 91.6% sensitivity and 98.9% specificity) in external validation studies.^5^ The NHIS did not conduct prospective follow-up, so we were not able to assess the clinical consequences of delayed insulin initiation.

Using the most recent national data available, we highlight an age-related risk for delayed insulin initiation among persons with adult-onset type 1 diabetes in the USA. Awareness and tools to improve classification and access to timely insulin treatment are needed in this growing patient population.

## References

[1] **Fang M, Wang D, Echouffo-Tcheugui JB, Selvin E.** Age at Diagnosis in US Adults With Type 1 Diabetes. Ann Intern Med. 2023;176(11):1567-1568. 37748184 10.7326/M23-1707 PMC10841362

[2] **Leslie RD, Evans-Molina C, Freund-Brown J, et al.** Adult-onset type 1 diabetes: current understanding and challenges. Diabetes Care. 2021;44(11):2449-2456. 34670785 10.2337/dc21-0770 PMC8546280

[3] **Lawrence JM, Slezak JM, Quesenberry C, et al.** Incidence and predictors of type 1 diabetes among younger adults aged 20–45 years: The diabetes in young adults (DiYA) study. Diabetes Res Clin Pract. 2021;171:108624. 33338552 10.1016/j.diabres.2020.108624 PMC10116767

[4] National Center for Health Statistics. 2022 National Health Interview Survey Description. 2023. https://ftp.cdc.gov/pub/Health_Statistics/NCHS/Dataset_Documentation/NHIS/2022/adult-codebook.pdf. Accessed 3 Aug 2023.

[5] **Nooney JG, Kirkman MS, Bullard KM, et al.** Identifying optimal survey-based algorithms to distinguish diabetes type among adults with diabetes. J Clin Transl Endocrinol. 2020;21:100231. 32695611 10.1016/j.jcte.2020.100231 PMC7365930

